# Metabolic heterogeneity‐based radiomic model predicts treatment outcomes of primary mediastinal B‐cell lymphoma patients in the IELSG37 study

**DOI:** 10.1111/bjh.70614

**Published:** 2026-06-16

**Authors:** L. Ceriani, L. Milan, L. Cascione, A. Di Rocco, I. Kryachok, A. J. Davies, A. Stathis, D. Rossi, P. W. M. Johnson, M. Martelli, E. Zucca

**Affiliations:** ^1^ Imaging Institute of Southern Switzerland ‐ EOC Lugano Switzerland; ^2^ Institute of Oncology Research Bellinzona Switzerland; ^3^ Università Della Svizzera Italiana Faculty of Biomedical Sciences Lugano Switzerland; ^4^ Division of Hematology, Department of Translational and Precision Medicine Sapienza University Rome Italy; ^5^ Oncohematology Department National Cancer Institute Kyiv Ukraine; ^6^ Cancer Research UK Centre, University of Southampton Southampton UK; ^7^ Oncology Institute of Southern Switzerland ‐ EOC Bellinzona Switzerland

**Keywords:** 18FDG‐PET/CT, PET metrics, PMBCL, prognostic models, radiomics

## Abstract

Early identification of patients at risk of failing first‐line therapy remains a key challenge in primary mediastinal B‐cell lymphoma (PMBCL). This study assessed whether positron emission tomography (PET) radiomics could predict treatment outcomes in PMBCL. Baseline PET/computed tomography (CT) scans from 501 patients enrolled in the International Extranodal Lymphoma Study Group 37 (IELSG37) trial and treated with rituximab‐ and doxorubicin‐based immunochemotherapy were analysed. Functional PET parameters reflecting tumour burden (metabolic tumour volume, MTV), glucose consumption (total lesion glycolysis, TLG) and metabolic heterogeneity (MH) were calculated using a 25% maximum standard uptake value (SUVmax) segmentation threshold. Radiomic analysis was performed in 495 patients, extracting 107 features; 74 features uncorrelated with SUVmax and MTV were selected and analysed using a recursive‐partitioning classification tree (Ctree) approach. Ctree identified grey‐level run‐length matrix run variance (GLRLM‐RV), a marker of MH, as the most significant prognostic feature. Patients with low MH (GLRLM‐RV <0.136) had a 5‐year progression‐free survival (PFS) of 93%, compared with 65% in those with high MH (*p* < 0.0001). Five‐year overall survival (OS) was 96% vs. 80% respectively (*p* < 0.0001). A subsequent Ctree model integrating GLRLM‐RV and TLG further improved prognostic stratification for PFS and OS (*p* < 0.0001), outperforming established prognostic indices (International Prognostic Index [IPI], revised‐IPI and age‐adjusted IPI).

## INTRODUCTION

Primary mediastinal large B‐cell lymphoma (PMBCL) is a subtype of diffuse large B‐cell lymphoma (DLBCL) that originates from B cells in the thymus.^1^, ^2^ It typically presents as a rapidly progressive, bulky mediastinal mass, often leading to local invasion and compressive syndromes. PMBCL predominantly affects young adults, most often white women, and is associated with a high cure rate when treated with aggressive immunochemotherapy regimens, with or without radiotherapy.^3^, ^4^, ^5^ Outcomes appear better than in other large B‐cell subtypes, partly because of the patients' younger age, but if the initial treatment fails, the results of standard second‐line chemotherapy are often poor.^6^, ^7^ Although recent therapeutic innovations such as Chimeric Antigen Receptor T (CAR‐T) cell‐therapy,^8^ bispecific antibodies,^9^ immune checkpoint inhibitors and antibody drug conjugates^10^ may improve patient outcomes after recurrence, the long‐term safety of initial treatment remains a paramount concern^11^ and the early identification of the small subset of patients likely to fail initial therapy could enable the development of risk‐adapted treatment strategies.

Currently, there is an unmet need for reliable prognostic markers in PMBCL, as the International Prognostic Index (IPI) offers limited predictive value. Positron emission tomography/computed tomography (PET/CT) using 18‐fluorodeoxyglucose (18FDG) has emerged as a promising tool for assessing tumour metabolic activity and stratifying risk in aggressive lymphomas.^12^ Baseline quantitative 18FDG‐PET/CT parameters, such as total lesion glycolysis (TLG), which reflects tumour glucose metabolism, and the area under the curve of the cumulative standardized uptake value (SUV) volume histogram (AUC‐CSH), an indicator of metabolic heterogeneity (MH), have shown strong prognostic value in the International Extranodal Lymphoma Study Group (IELSG) 26 study.^13^, ^14^, ^15^ Notably, a simple prognostic model based on TLG and MH resulted in early and accurate identification of patients at high risk for progression.^15^

Recent advances in radiomics have enabled the extraction of additional quantitative features from PET images, which may serve as novel biomarkers across various lymphoma subtypes.^16^ These mathematical and statistical features describe lesion texture, radiotracer uptake intensity, tumour heterogeneity and shape, potentially reflecting underlying biological characteristics such as proliferation, hypoxia, necrosis and angiogenesis.^17^, ^18^, ^19^, ^20^ This study aims to assess whether baseline PET radiomics can refine patient stratification in PMBCL to facilitate the implementation of tailored therapeutic strategies. By identifying individuals with an elevated risk of treatment failure, these quantitative parameters may inform personalized management approaches and ultimately enhance clinical outcomes.

## METHODS

### Ethics Statement

The IELSG37 clinical trial of the IELSG was conducted in accordance with the precepts of the 1964 Helsinki Declaration and its later amendments. The study was approved by the Institutional Review Board/Ethics Committee of each participating centre and all patients gave written informed consent. This trial was registered at www.clinicaltrials.gov as #NCT01599559.

### Patient population

Among the 545 patients with untreated PMBCL prospectively enrolled in the IELSG37 trial from 43 centres, 501 with baseline 18FDG‐PET/CT scans suitable for analysis and a complete clinical follow‐up were eligible for this study. All patients received rituximab and doxorubicin‐based immunochemotherapy regimens (Table S1), and 342 of them underwent consolidation radiotherapy.^3^, ^4^

The International Prognostic Index (IPI), revised‐IPI (R‐IPI) and the age‐adjusted IPI (aaIPI) were calculated for each patient.^21^, ^22^

### 18FDG PET/CT imaging

PET‐CT scans using 18FDG were performed at baseline within 14 days of starting treatment. The PET‐CT images were performed according to the guidelines of the European Association of Nuclear Medicine version 2.0.^23^ Specifically, PET scans were acquired from the mid‐thigh to the base of the skull 60 ± 10 min after injecting 3–4.5 MBq/kg of 18FDG. Patients were asked to fast for at least 6 h, and their blood glucose had to be <160 mg/dL before injecting the radiotracer. The CT scans were obtained with a low‐dose protocol and used for attenuation‐corrected PET image reconstruction.

PET‐CT centres had to meet the requirements for clinical trial qualification, which were assessed by the imaging core labs of the National Cancer Research Institute based at St Thomas' Hospital, London, for the UK and Norway, and the Medical Physics Unit of Santa Croce e Carle Hospital, Cuneo, Italy, for all other countries. Each centre was required to document an active quality assurance/control programme and apply data acquisition and reconstruction protocols validated by the core labs under the clinical trial qualification programme. Participating PET centres uploaded anonymized PET‐CT scans onto a web‐based system.^24^ Before further analysis, a medical physicist at the core labs performed a technical review of the scans to ensure satisfactory data quality and adherence to the protocol.

### PET/CT images analysis and radiomic features extraction

All scans were centrally evaluated using standard imaging software (MM Oncology, Syngo.Via VB60A HF05, Siemens) on a dedicated workstation. The software automatically segmented all tumour lesions for each patient using a fixed 25% maximum SUV (SUVmax) threshold to estimate metabolic tumour volume (MTV), a method specifically validated for PMBCL.^13^, ^14^, ^25^ This approach has shown comparable performance to the more recently proposed standardized method based on an SUV ≥4 threshold.^26^ SUVmax, peak standard uptake value (SUVpeak) and TLG were measured automatically. The segmentation results were visually checked by an expert nuclear physician and manually corrected if necessary.

The 3D contoured map was generated and exported for further radiomic analysis of the tumour volume in 495 of the 501 baseline PET studies. In six cases, this could not be obtained due to technical problems. A total of 107 3D standardized radiomic features (RFs) evaluating different characteristics of the tumour lesions were extracted from each PET/CT study using the PyRadiomics open‐source Python package (version 2.2.0).^27^ To standardize this process, the grey‐level intensities and voxel dimensions of the original images were resampled following the Image Biomarkers Standardisation Initiative (IBSI)^28^; details of the extraction settings (Table S2) and the complete list of features (Table S3) are provided in the Supplement.

Each RF was first standardized using the *Z*‐score with $Z=\left(\mu -\bar{\mu }\right)/\sigma$, where *μ*, $\bar{\mu }$ and *σ* are the value, the mean and the standard deviation respectively. Considering RF redundancy, we used Spearman correlation test (*p* < 0.05) with false discovery rate (FDR) correction to reduce the overfitting problem and to discard features highly correlated to SUVmax and MTV (correlation coefficient >0.7).^29^

### Statistics

Continuous variables were expressed as median and interquartile range (IQR) and their distribution between groups was compared using the Mann–Whitney *U*‐test. Differences between the frequencies of categorical data were assessed with the chi‐square test or the Fisher exact test, as applicable. The PET‐associated functional continuous parameters were analysed as dichotomized variables, using receiver‐operating characteristic (ROC) analysis to identify the optimal cut‐off point to discriminate subgroups with different PFS and overall survival (OS). PFS was defined as the time from study entry until lymphoma progression or death from any cause; OS was defined as the time from entry to death from any cause.^30^ Survival curves were generated using the Kaplan–Meier method and compared using the log‐rank test (or the log‐rank test for trend, as appropriate). Multivariable analysis and estimation of hazard ratio (HR) were conducted using Cox proportional hazard models.

We built the classification tree using a recursive‐partitioning classification tree (Ctree) method (implemented into the ‘Ctree’ function of the R package party) to develop unbiased prognostic models based on dichotomized variables. This procedure enables the hierarchical classification of the prognostic covariates, from the most important, which splits the primary node (entire population), to those which extend to the terminal nodes (risk groups).^31^

The predictive accuracy of the different prognostic models was compared by concordance probability estimate (CPE), using Harrell's C statistic.^32^ The indices' relative quality was further assessed using the Akaike information criterion (AIC), an in‐sample fit approach to model selection.^33^ A *p*‐value <0.05 was considered statistically significant. Statistical analyses were conducted using the STATA 17.1 statistical software (StataCorp) and the RStudio 1.2.1335 software package (RStudio, Posit Public Benefit Corporation (PBC)) with R version 4.0.3, as appropriate.

## RESULTS

Five hundred and one of the 545 patients enrolled in the IELSG37 study (92%) had baseline PET scans and suitable clinical data. Their median age at study entry was 34 years (IQR, 28–44 years). Table 1 presents the main clinical characteristics of the patient cohort. Baseline functional PET parameters for the study population are summarized in Table 2. After a median follow‐up of 64 months (IQR, 48–69 months), 37 patients experienced disease progression, and 25 patients died; in only 5 cases was the cause of death unrelated to lymphoma or its treatment.

**TABLE 1:** Clinical features at presentation (*N* = 501).

Characteristic (*n* = 501)	No. of patients (%)
Age ≤60 years	481 (96)
Female sex	302 (60)
ECOG Performance status 0–1	460 (92)
Bulky mediastinal lesion (>10 cm)	356 (71)
Bone marrow involvement	2 (0.4)
Ann Arbor stage I‐II	489 (98)
LDH >ULN	355 (71)
IPI	
Low risk	309 (62)
Low‐intermediate risk	163 (32)
Intermediate‐high risk	28 (5.5)
High risk	1 (0.5)
Revised IPI	
Low risk	97 (19)
Intermediate risk	376 (75)
High risk	28 (6)
Age‐adjusted IPI (*n* = 481)	
Low risk	132 (26)
Low‐intermediate risk	308 (61.5)
Intermediate‐high risk	39 (8)
High risk	2 (0.5)

Abbreviations: ECOG, Eastern Cooperative Oncology Group; IPI, International Prognostic Index; LDH, serum lactate dehydrogenase; ULN, upper limit of normal.

**TABLE 2:** Baseline 18FDG PET‐CT parameters.

Parameter	Median	Interquartile range
SUVmax	21.5	17.6–26.7
SUVpeak	18.3	15.1–22.6
MTV (mL)	331	201–530
TLG	3858	1894–6070
AUC‐CSH	0.476	0.431–0.516
Lesion diameter (mm)	120	97–140
GLRLM‐RV	0.0720	0.0589–0.0905

Abbreviations: AUC‐CSH, area under the curve of the cumulative standardized uptake value (SUV)–volume histogram; GLRLM‐RV, grey‐level run length matrix run variance; MTV, metabolic tumour volume; SUVmax, maximum standard uptake value; SUVpeak, peak standard uptake value; TLG, total lesion glycolysis.

### Radiomic analysis

RF analysis was possible in only 495 patients and was conducted on the 107 RFs originally extracted. Out of these, 32 were found to be highly correlated to SUVmax or MTV and were therefore removed from further analysis (detailed list in Table S3). The remaining 75 RFs were then analysed using an unsupervised recursive partitioning Ctree algorithm.

**FIGURE 1:**
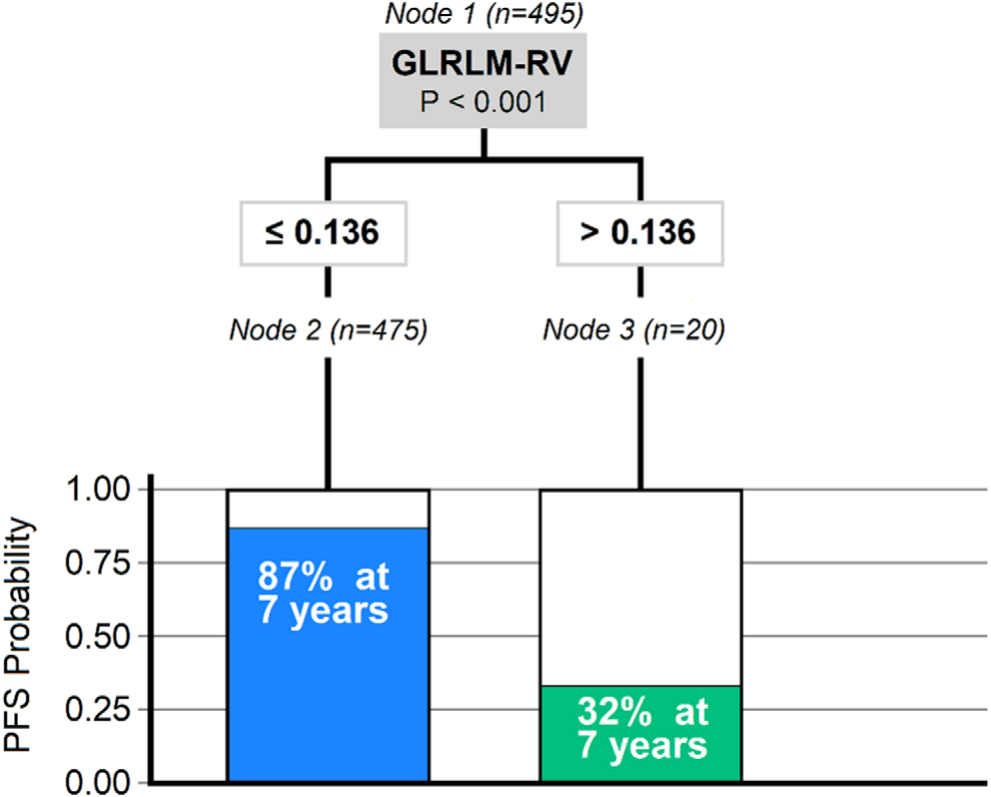
Long‐term impact of grey‐level run length matrix run variance (GLRLM‐RV) on progression‐free survival (PFS) in the Ctree model resulting from the analysis of 75 radiomic features selected for their lack of correlation with maximum standard uptake value (SUVmax) or metabolic tumour volume (MTV).

**FIGURE 2:**
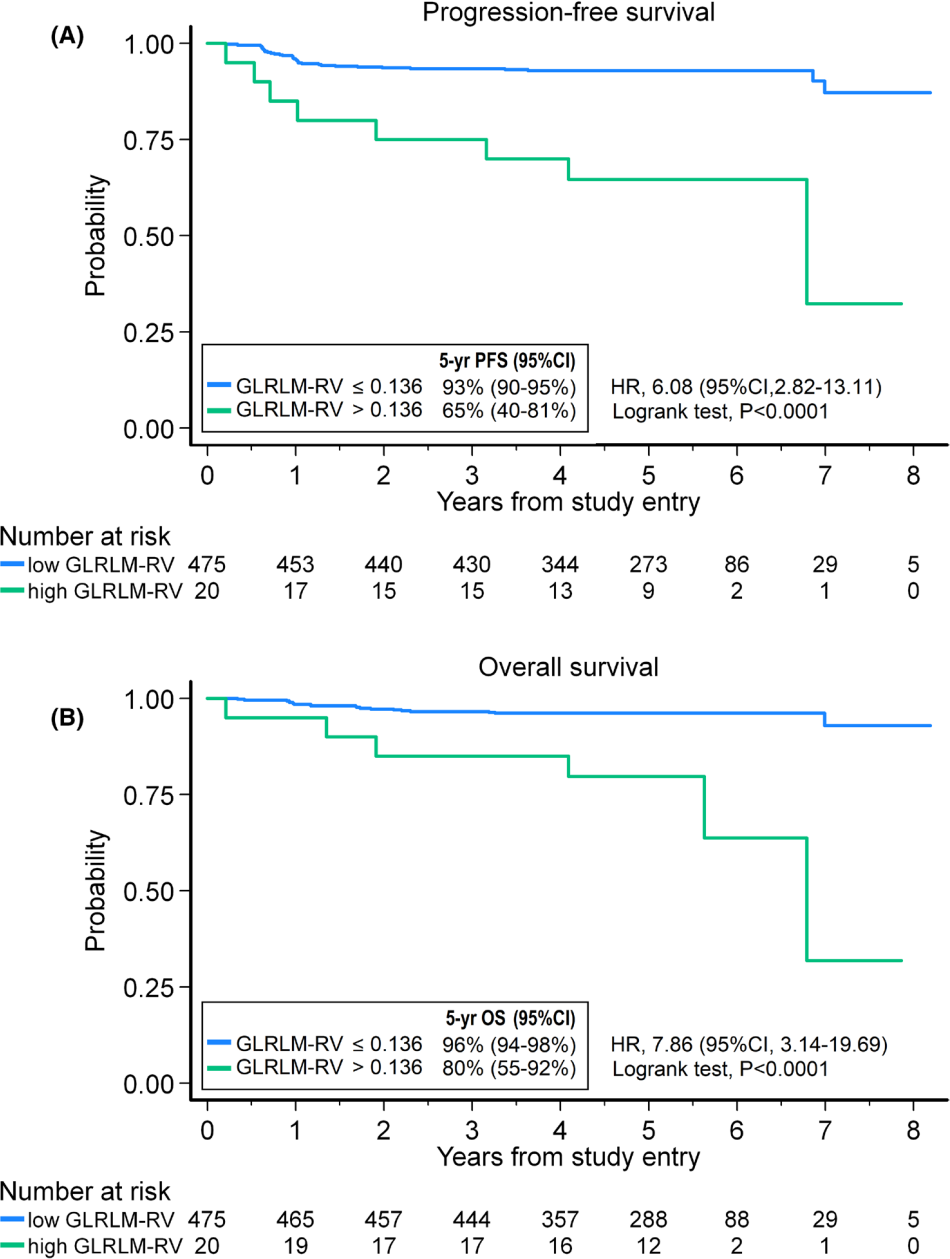
Kaplan–Meier estimates of progression‐free survival (PFS, panel A) and overall survival (OS, panel B) according to the grey‐level run length matrix run variance (GLRLM‐RV) values.

The grey‐level run‐length matrix run variance (GLRLM‐RV), see Supplemental Data for its mathematical definition, a parameter describing the MH of the lesions, was identified as the strongest predictor of PFS (*p* < 0.001; Figure 1). The subgroup of patients with GLRLM‐RV value higher than the Ctree‐generated threshold (0.136) had worse 5‐year PFS compared to those with lower values (65% vs. 93%, *p* < 0.0001; with a HR of 6.1; Figure 2A). Moreover, this RF was able to discriminate patients with different OS rates (80% at 5 years for those with high values vs. 96% for those with low values; HR, 7.9; *p* < 0.0001; Figure 2B).

### Univariable analysis of clinical and PET metrics prognostic factors

The ROC curve analysis that generated the PET metrics cut‐points is summarized in the supplemental data (Table S4). At univariable analysis of baseline clinical features and PET parameters, sex, stage, largest lesion diameter and number of extra‐nodal localizations as well as SUVmax, SUVpeak, MTV and TLG were significantly associated with PFS (Table 3).

**TABLE 3:** Univariable analysis of progression‐free survival (PFS) according to dichotomized baseline clinical and PET parameters (*N* = 501).

Baseline parameters	No. of patients	5‐year PFS (95% CI)	HR (95% CI)	*p* value (log‐rank test)
Bulk				
≤100 mm	145	95% (90%–98%)	2.07 (0.92–4.66)	0.0728
>100 mm	356	91% (87%–93%)		
Largest lesion diameter				
≤139.5 mm	368	94% (91%–96%)	2.55 (1.40–4.64)	**0.0015**
>139.5 mm	133	86% (79%–91%)		
Sex				
Male	199	89% (84%–93%)	0.51 (0.28–0.94)	**0.0272**
Female	302	94% (90%–96%)		
Age				
≤60 year	481	92% (89%–94%)	0.62 (0.08–4.53)	0.6359
>60 year	20	95% (69%–99%)		
No. of extra‐nodal sites				
≤1	310	94% (90%–96%)	1.87 (1.02–3.40)	**0.0386**
>1	191	89% (83%–92%)		
LDH				
≤ULN	146	92% (86%–96%)	1.19 (0.60–2.37)	0.6101
>ULN	355	92% (88%–94%)		
Ann Arbor stage^a^				
1–2	489	92% (90%–95%)	5.95 (2.12–16.72)	**0.0001**
3–4	12	67% (34%–86%)		
ECOG PS				
≤1	460	92% (90%–95%)	2.14 (0.94–4.83)	0.0620
>1	41	85% (70%–93%)		
TLG				
≤3858	289	96% (93%–98%)	3.70 (1.90–7.21)	**<0.0001**
>3858	212	86% (81%–90%)		
MTV				
≤283 mL	208	96% (92%–98%)	3.26 (1.51–7.02)	**0.0014**
>283 mL	293	89% (85%–92%)		
AUC‐CSH				
≥0.463	291	93% (89%–95%)	1.35 (0.74–2.45)	0.3253
<0.463	210	91% (86%–94%)		
SUVmax				
≤23.4	305	94% (91%–96%)	1.82 (1.00–3.31)	**0.0467**
>23.4	196	89% (83%–92%)		
SUVpeak				
≤21.2	339	94% (91%–96%)	2.03 (1.11–3.69)	**0.0181**
>21.2	162	87% (81%–92%)		
GLRLM‐RV				
≤0.136	475	93% (90%–95%)	6.08 (2.82–13.11)	**< 0.0001**
>0.136	20	65% (40%–81%)		

*Note*: Bolded values indicate *p*<0.05, representing a statistically significant correlation between the parameter and PFS.

Abbreviations: 95% CI, 95% confidence interval; AUC‐CSH, area under the curve of the cumulative standardized uptake value (SUV)–volume histogram SUVmax, maximum SUV; ECOG PS, Eastern Cooperative Oncology Group performance status; GLRLM‐RV, grey‐level run length matrix run variance; HR, hazard ratio; LDH, serum lactate dehydrogenase; MTV, metabolic tumour volume; SUVpeak, peak value of SUV; TLG, total lesion glycolysis; ULN, upper limit of normal.

^a^ Patients with advanced stage disease were not eligible in the IELSG37 trial; however, 10 patients with contiguous nodal involvement extending below the diaphragm and 2 patients with single focal bone marrow uptake were also enrolled.

### Prognostic model generation

**FIGURE 3:**
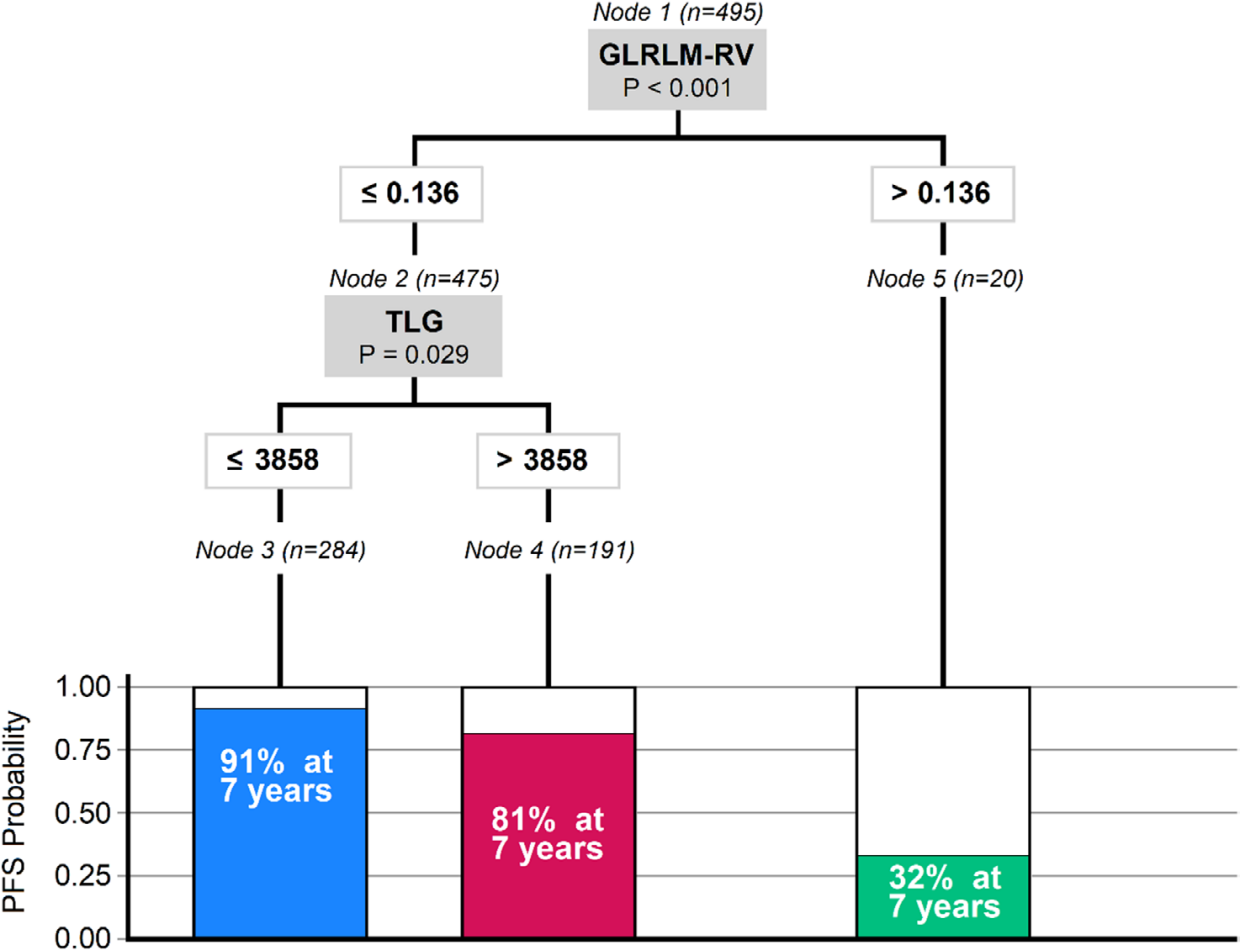
Long‐term impact on progression‐free survival (PFS) according to the C‐tree model that integrates grey‐level run length matrix run variance (GLRLM‐RV) and total lesion glycolysis (TLG).

The Ctree algorithm was then applied to generate a prognostic model by adding to GLRLM‐RV the above‐mentioned baseline clinical and PET features with a significant impact on PFS in univariable analysis. The algorithm generated a conditional inference tree with three terminal nodes using GLRLM‐RV and TLG as predictive variables, as depicted in Figure 3. An internal validation of the C‐tree model based on bootstrap analysis confirmed GLRLM‐RV and TLG as the dominant PFS predictors (data not shown). Notably, GLRLM‐RV and TLG remained independent significant outcome predictors in a multivariable analysis (Table S5).

According to the decision tree model, in the first node, GLRLM‐RV identified a partition of the initial population into two subgroups based on the degree of MH. Patients with high MH had a lower PFS than those with low MH. On the other hand, patients with homogeneous FDG uptake (low MH) were stratified into two subgroups based on their TLG values. Specifically, patients with a TLG value greater than 3858 had lower PFS than those with low TLG.

**FIGURE 4:**
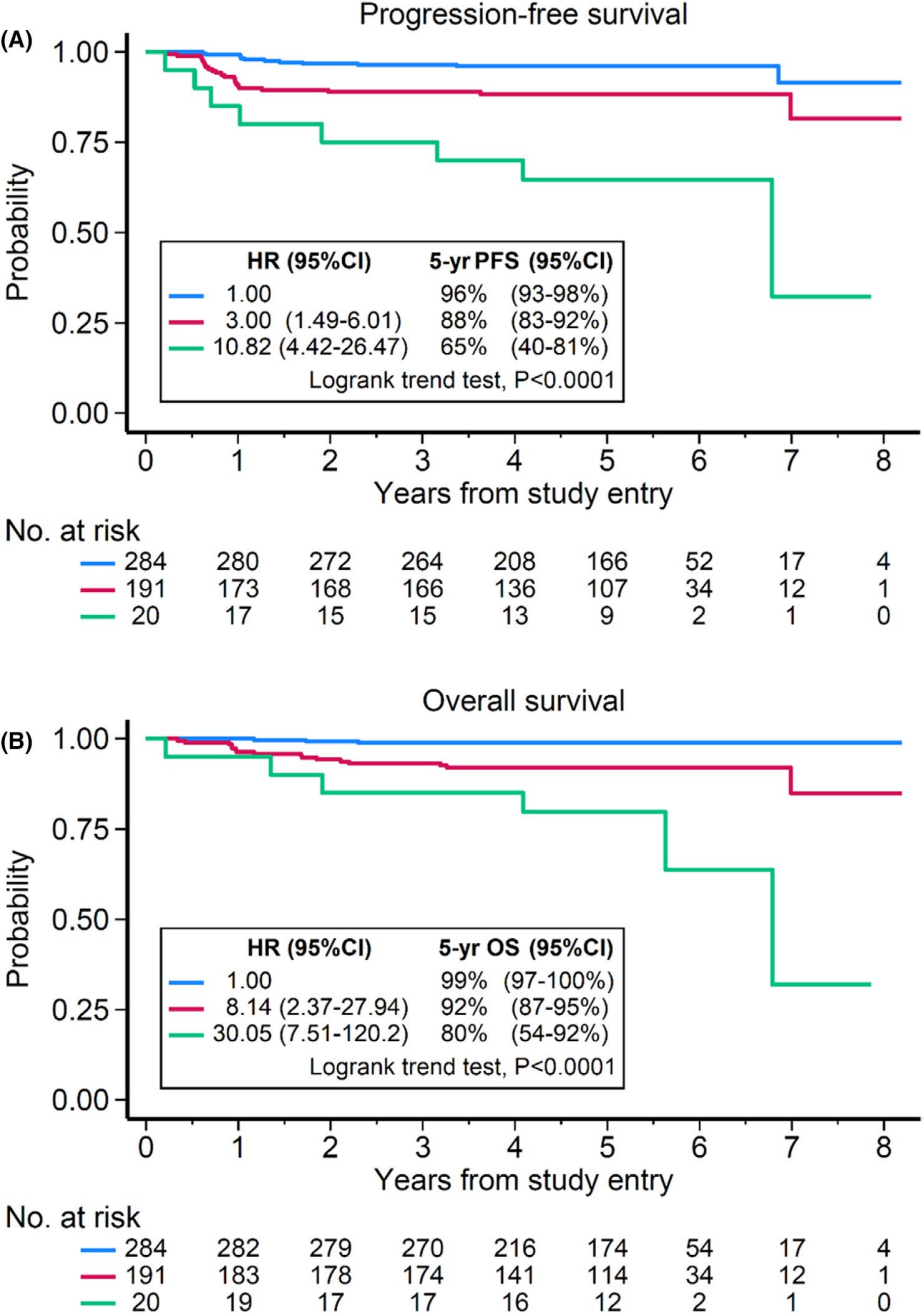
Kaplan–Meier estimates of progression‐free survival (panel A) and overall survival (panel B) according to the Ctree‐derived PET radiomic model. The model separates three patient prognostic groups: A very good risk group (defined by low grey‐level run length matrix run variance [GLRLM‐RV] and low total lesion glycolysis [TLG], blue line), a standard risk group (defined by low GLRLM‐RV and high TLG, red line), and a poor risk group (defined by high GLRLM‐RV with any TLG, green line).

The three groups had different 5‐year PFS (62% for the high‐risk group, 88.0% for the intermediate‐risk group, and 96% for the low‐risk group; log‐rank test trend *p* < 0.0001). The model based on the integration of MH and TLG maintained the same predictive value for progression or relapse in the high‐risk group and increased the Negative Predictive Value (NPV) in the low‐risk group compared to GLRLM‐RV alone (Figure 4A).

Although the model was generated based on features prognostic for PFS, it was also able to predict OS, with a significantly different risk of death across the same three subgroups. Patients with high MH and any TLG value had a 5‐year OS of 80%, those with low MH and high TLG had a 5‐year OS of 92%, and those with low MH and low TLG had a 5‐year OS of 99% (log‐rank test for trend, *p* < 0.0001) (Figure 4B). MH and TLG were also the individual factors most strongly associated with outcome in the univariable and multivariable analysis of OS (Tables S5 and S6).

### Comparison between prognostic models

A concordance probability analysis demonstrated that this model (Harrell C 0.67 for PFS and 0.69 for OS) improved the prognostic capacity over either the TLG (Harrell C 0.64 for PFS and 0.68 for OS) or the GLRLM‐RV alone (Harrell C 0.62 for both PFS and OS). Furthermore, the model was compared with standard IPIs. The performance of various prognostic models assessed using either the Akaike information criterion or Harrell C concordance probability index, the statistic, is summarized in the supplemental material (Table S7). The radiomic model demonstrated superior discriminatory power and predictive accuracy for both PFS and OS over the IPI, R‐IPI and age‐adjusted IPI. This radiomic model also outperformed the model previously proposed from the analysis of the IELSG26 study,^15^ which was based on TLG and the AUC‐CSH value, a simpler marker of MH.

## DISCUSSION

This study demonstrated that radiomics derived from staging PET/CT may play an important role in predicting the outcome of PMBCL patients treated with conventional immunochemotherapy. It also confirmed the potential clinical relevance of MH in this lymphoma subtype.

In the IELSG 26 study, we previously showed that MH, estimated using the AUC‐CSH method, could better characterize mediastinal masses, especially those with higher TLG. Specifically, we showed that combining the intralesion distribution of glucose metabolism with TLG may provide a better marker of biological aggressiveness compared to TLG alone.^15^

In this larger patient cohort, radiomics demonstrated superior performance in assessing MH compared with the AUC‐CSH method. Because radiomic textural analysis yields a three‐dimensional characterization of MH, it more accurately reflects the intralesional distribution of glucose metabolism than the simple statistical description obtained with AUC‐CSH.

In our cohort, GLRLM‐RV was the strongest PFS predictor among the radiomic texture parameters. The predictive value of MH in large masses is also consistent with findings in DLBCL. In a post‐hoc analysis of the SAKK38/07 trial, we showed that MH may represent an important disease characteristic with prognostic significance in patients with a high metabolic tumour burden.^34^ The Ctree‐generated prognostic model indicated that combining GLRLM‐RV and TLG can allow fine tuning of patient risk, which is consistent with the prior IELSG26 results.^15^

This study also confirms previous observations showing that, unlike Hodgkin lymphoma and DLBCL, the prognostic value of MTV in PMBCL is lower than that of TLG.^13^, ^35^ In addition, it provides new and robust evidence supporting the prognostic value of PET‐derived radiomics in PMBCL. Several preliminary studies have explored this issue in other lymphoma subtypes, particularly DLBCL, although variations in the methods used for RF extraction have led to inconsistent results. With the exception of MTV, the prognostic application of radiomics in B‐cell lymphomas remains poorly understood.

In PMBCL, baseline PET radiomics has been proposed as a tool for differential diagnosis from Hodgkin lymphoma and grey zone lymphomas, which often present with large mediastinal masses,^36^ although without demonstrated prognostic relevance. Conversely, a Canadian research group used a machine learning approach to evaluate changes in radiomic parameters and identified them as potential predictors of outcomes in PMBCL.^37^ More recently, Esposito et al. developed and validated a machine learning model that uses 18F‐FDG PET/CT RF to predict response to frontline therapy in a small cohort of PMBCL patients.^38^

Our model, developed from the largest cohort of PMBCL patients prospectively enrolled in a multicentre clinical trial, demonstrated that radiomic analysis of baseline PET imaging can provide robust information for the early identification of high‐risk patients with shorter PFS, as well as very good‐risk patients with near‐100 percent chances of cure. It has the potential to inform treatment strategies but should be prospectively evaluated in future clinical trials. Although derived using PFS, the same model also showed an association with OS; however, given the limited number of OS events relative to the number of candidate RF, some risk of overfitting cannot be excluded. Therefore, the OS findings should be considered exploratory and supportive until validated in independent cohorts.

## AUTHOR CONTRIBUTIONS

**L. Ceriani:** Conceptualization; funding acquisition; validation; formal analysis; writing – original draft; writing – review and editing. **L. Milan:** Conceptualization; formal analysis; data curation; writing – review and editing. **A. Di Rocco:** Investigation; writing – review and editing. **L. Cascione:** Validation; formal analysis; data curation; software; visualization; writing – original draft; writing – review and editing. **E. Zucca:** Conceptualization; investigation; funding acquisition; writing – original draft; writing – review and editing; formal analysis; validation; visualization; methodology; data curation; supervision; resources; project administration. **I. Kryachok:** Writing – review and editing; investigation. **D. Rossi:** Investigation; writing – review and editing. **P. W. M. Johnson:** Investigation; writing – review and editing. **M. Martelli:** Conceptualization; investigation; writing – original draft; writing – review and editing; validation; methodology; visualization; supervision. **A. J. Davies:** Conceptualization; investigation; writing – review and editing; funding acquisition. **A. Stathis:** Investigation; writing – review and editing.

## FUNDING INFORMATION

The IELSG37 trial was funded by grants from the Swiss National Science Foundation (SNSF Project 32003B_146931), Swiss Cancer Research foundation (KFS‐2852‐08‐2011), Cancer Research UK (A16247 CRUK/12/040), National Institute for Health and Care Research (RP‐2016‐07‐001/NIHR) and Rising Tide Foundation for Clinical Cancer Research (CCR‐22‐110). The present analysis was supported by a grant from the Swiss Cancer Research foundation (KLS‐5406‐08‐2021).

## CONFLICT OF INTEREST STATEMENT

The authors have no conflicts of interest to disclose.

## Supporting information


**Table S1.** Front‐line immunochemotherapy regimens administered to the 501 patients enrolled in the IELSG37 study and included in the present radiomic analysis. In addition, 342 of them also received consolidation radiotherapy.
**Table S2.** Parameters used to extract radiomic features.
**Table S3.** List of 107 extracted radiomic features; the 75 included in the *Ctree* model are shown in bold.
**Table S4.** Results of ROC curve analysis and dichotomization of baseline 18FDG PET parameters.
**Table S5.** Multivariable Cox models for progression‐free and overall survival.
**Table S6.** Univariate analysis of overall survival according to dichotomized baseline clinical and PET parameters (*N* = 501).
**Table S7.** Model performance for progression‐free (PFS) and overall survival (OS) prediction.

## Data Availability

The clinical trial dataset generated and analysed during this study is not publicly available due to legal restrictions. De‐identified data sharing is possible only for scientific research on a reasonable request. Data sharing requests should be addressed to the International Extranodal Lymphoma Study Group.
