# Supplementary material for: Metabolic heterogeneity‐based radiomic model predicts treatment outcomes of primary mediastinal B‐cell lymphoma patients in the IELSG37 study
Source: Br J Haematol. 2026 Jun 16;209(2):514–23. doi: 10.1111/bjh.70614 (PMC13462169; doi:10.1111/bjh.70614)
Supplement: Supplementary file 1 [file BJH-209-514-s001.docx]

***Supplemental data***

**Table S1**. Front-line immunochemotherapy regimens administered to the 501 patients enrolled in the IELSG37 study and included in the present radiomic analysis. In addition, 342 of them also received consolidation radiotherapy

| Immunochemoterapy regimen (n=501) | No. of patients  (%) |
| --- | --- |
| R-VACOP/MACOP-B | 160 (32) |
| R-CHOP14 | 135 (27) |
| DA-EPOCH-R | 78 (16) |
| R-CHOP21 | 74 (14) |
| Others * | 54 (11) |

*Other regimens included R-Mega-CHOP, R-COMP, R-CHOEP, GMALL-B-ALL/NHL200

**Radiomics Feature Extraction Methodology**

Radiomics features (RF) were extracted from PET/CT images using the PyRadiomics platform, a software package compliant with the Image Biomarker Standardization Initiative (IBSI). PyRadiomics is a flexible, open-source Python-based tool capable of extracting a large set of engineered features from medical images. These features are subdivided into the following classes:

- First-Order Statistics (19 features)
- Shape-Based (3D) (16 features)
- Shape-Based (2D) (10 features)
- Gray Level Co-occurrence Matrix (GLCM) (24 features)
- Gray Level Run Length Matrix (GLRLM) (16 features)
- Gray Level Size Zone Matrix (GLSZM) (16 features)
- Neighboring Gray Tone Difference Matrix (NGTDM) (5 features)
- Gray Level Dependence Matrix (GLDM) (14 features)

All feature classes, except for the shape-based ones, were computed on both the original image and on derived images obtained by applying various filters. Shape descriptors are independent of gray-level intensity and represent the geometric properties of the lesion. First-order statistical features describe areas with different metabolic activity levels without considering their spatial distribution, whereas matrix-based features capture the spatial arrangement and interrelationships of these regions.

Table S1 lists the default parameters used for RF extraction, and Table S2 enumerates the 107 extracted RF. Spearman’s rank correlation coefficient with false discovery rate (FDR) correction was used to remove features highly correlated (ρ > 0.7) with tumor uptake (SUVmax) or metabolic tumor volume (MTV). Seventy-five uncorrelated RF were retained for inclusion in the Ctree analysis, they comprise 6 first-order features, 6 3D shape features, and 63 second-order matrix features. Their definitions, as specified by the Image Biomarker Standardisation Initiative (IBSI), are available at [https://ibsi.readthedocs.io/en/latest/03_Image_features.html#](https://ibsi.readthedocs.io/en/latest/03_Image_features.html) and their mathematical descriptions are provided in the PyRadiomics online documentation at [https://pyradiomics.readthedocs.io/en/latest/features.html#](https://pyradiomics.readthedocs.io/en/latest/features.html).

**GLRLM-RV calculation**

Gray Level Run Length Matrix (GLRLM): A run length is defined as the length of a consecutive sequence of voxels with the same grey level along direction ϑ. By default, the value of a feature is calculated on the GLRLM for each angle separately, after which the mean of these values is returned. The GLRLM then contains the occurrences of runs with length *j* for a discretised grey level *i* [IBSI].

Run Variance is of the variance in runs for the run lengths. This feature estimates the variance in runs over the run lengths.

$$Run Variance= \sum_{i=1}^{N_{g}} \sum_{j=1}^{N_{r}} p\left( i,j | \vartheta\right)\left( i- \mu\right)^{2}$$

where $p\left( i,j | \vartheta\right)$ is the normalized run length matrix and µ is the mean run length:

$$\mu=\sum_{i=1}^{N_{g}} \sum_{j=1}^{N_{r}} p\left( i,j | \vartheta\right)j$$

**Table S2:** Parameters used to extract radiomics features

| **Calculation setting** | **Default** |
| --- | --- |
| Approach | 3D |
| Interpolation   - Voxel dimension (mm) - Interpolation method - ROI interpolation method | Yes   - 3x3x3 - B-Spline - Nearest-Neighbor |
| Resegmentation   - Range (SUV) - Outliers | - No - No |
| Discretization | Fixed bin size: 64 bins |

ROI, region of interest; SUV, standardized uptake value.

**Table S3:** List of 107 extracted radiomic features; the 75 included in the *Ctree* model are shown in bold.

| **First-order features** | | | | |  |
| --- | --- | --- | --- | --- | --- |
| *Histogram based features* | | | | |  |
| 10Percentile  90Percentile  Energy  **Entropy***  InterquartileRange  **Kurtosis***  **Maximum***  MeanAbsoluteDeviation  Mean  Median  **Minimum***  Range  RobustMeanAbsoluteDeviation  RootMeanSquared  **Skewness***  TotalEnergy  **Uniformity***  Variance | | | | |  |
| **Shape-based features** | | | | | |
| *3D shape features* | | | | | |
| **Elongation***  **Flatness***  LeastAxisLength  **MajorAxisLength***  Maximum2DDiameterColumn  Maximum2DDiameterRow  Maximum2DDiameterSlice  **Maximum3DDiameter***  MeshVolume  MinorAxisLength  **Sphericity***  SurfaceArea  SurfaceVolumeRatio  **VoxelVolume*** | | | | | |
| **Second-order (texture) features** | | | | | |
| *GLCM* | *GLDM* | *GLRLM* | *GLSZM* | *NGTDM* | |
| **Autocorrelation***  **ClusterProminence***  **ClusterShade***  **ClusterTendency***  **Contrast***  **Correlation***  **DifferenceAverage***  **DifferenceEntropy***  **DifferenceVariance***  **Id***  **Idm***  **Idmn***  **Idn***  **Imc1***  **Imc2***  **InverseVariance***  **JointAverage***  **JointEnergy***  **JointEntropy***  **MCC***  **MaximumProbability***  **SumAverage***  **SumEntropy***  **SumSquares*** | **DependenceEntropy***  DependenceNonUniformity  **DependenceNonUniformityNormalized***  **DependenceVariance***  GrayLevelNonUniformity  **GrayLevelVariance***  **HighGrayLevelEmphasis***  **LargeDependenceEmphasis***  **LargeDependenceHighGrayLevelEmphasis***  **LargeDependenceLowGrayLevelEmphasis***  **LowGrayLevelEmphasis***  **SmallDependenceEmphasis***  **SmallDependenceHighGrayLevelEmphasis***  SmallDependenceLowGrayLevelEmphasis | GrayLevelNonUniformity  **GrayLevelNonUniformityNormalized***  **GrayLevelVariance***  **HighGrayLevelRunEmphasis***  **LongRunEmphasis***  **LongRunHighGrayLevelEmphasis***  **LongRunLowGrayLevelEmphasis***  **LowGrayLevelRunEmphasis***  **RunEntropy***  RunLengthNonUniformity  **RunLengthNonUniformityNormalized***  **RunPercentage***  **RunVariance***  **ShortRunEmphasis***  **ShortRunHighGrayLevelEmphasis***  **ShortRunLowGrayLevelEmphasis*** | GrayLevelNonUniformity  **GrayLevelNonUniformityNormalized***  **GrayLevelVariance***  **HighGrayLevelZoneEmphasis***  **LargeAreaEmphasis***  **LargeAreaHighGrayLevelEmphasis***  **LargeAreaLowGrayLevelEmphasis***  **LowGrayLevelZoneEmphasis***  SizeZoneNonUniformity  **SizeZoneNonUniformityNormalized***  **SmallAreaEmphasis***  **SmallAreaHighGrayLevelEmphasis***  **SmallAreaLowGrayLevelEmphasis***  ZoneEntropy  ZonePercentage  **ZoneVariance*** | Busyness  Coarseness  **Complexity***  **Contrast***  Strength | |

**Univariable and multivariable analysis of prognostic factors**

**Table S4** summarizes the results of ROC analysis for the dichotomization of baseline 18FDG PET parameters.

**Table S5** shows the final multivariable Cox proportional hazards regression models for PFS and OS obtained by backward stepwise selection from dichotomised variables identified as statistically significant at univariable analysis.

**Table S6** shows the results of univariable analysis (log-rank test) of overall survival according to dichotomized baseline clinical and PET parameters.

**Tables S7** shows the comparison of the accuracy and quality of different prognostic models for either progression-free survival or overall survival. The Harrel’s C concordance index (C-index) measures the rank correlation between predicted risk scores and observed event times. It ranges from 0.5 (random chance) to 1.0 (perfect prediction). Higher values indicate better models in terms of their ability to discriminate between high and low-risk individuals. AIC (Akaike Information Criterion) and BIC (Bayesian Information Criterion) are widely used statistical measures for model selection that balance model fit and complexity. Both criteria penalize models with more parameters to avoid overfitting and the model with the lower score is favored. AIC is less stringent and preferred for predictive accuracy. In contrast, BIC ends to select simpler models and is favored for identifying the "true" model in larger datasets.

**Table S4.** Results of ROC curve analysis and dichotomization of baseline 18FDG PET Parameters -

| Parameter | Progression-free Survival | | | | |  | Overall Survival | | | | |
| --- | --- | --- | --- | --- | --- | --- | --- | --- | --- | --- | --- |
|  | ***AUC***  ***(95% CI)*** | ***P-value*** | ***Cut-off**** | ***Sensitivity***  ***(95% CI)*** | ***Specificity***  ***(95% CI)*** |  | ***AUC***  ***(95% CI)*** | ***P-value*** | ***Cut-off**** | ***Sensitivity***  ***(95% CI)*** | ***Specificity***  ***(95% CI)*** |
| SUVmax | 0.55 (0.51 -0.60) | 0.251 | 23.4 | 53.5%  (37.7 - 8.8) | 62.2%  (57.6 - 6.7) |  | 0.59  (0.49 – 0.70) | 0.079 | 24.3 | 52%  (31.3 - 72.2) | 66.4%  (61.9 - 70.6) |
| SUVpeak | 0.57 (0.53 - 0.62) | 0.110 | 21.2 | 48.8%  (33.3 - 4.5) | 69.2%  (64.8 - 3.4) |  | 0.61  (0.51 - 0.71) | 0.034 | 16.1 | 92%  (74.0 - 99.0) | 33%  (28.8 - 37.4) |
| MTV (ml) | 0.64  (0.59 - 0.68) | 0.0014 | 283 | 81.4%  (66.6 - 1.6) | 43.7%  (39.1 - 8.3) |  | 0.73  (0.63 - 0.82) | <0.0001 | 318 | 88%  (68.8 - 97.5) | 50.2%  (45.6 - 54.8) |
| TLG | 0.65  (0.60 - 0.69) | 0.0007 | 3858 | 72.1%  (56.3 - 4.7) | 60.5%  (56.3 - 4.7) |  | 0.75  (0.67 - 0.83) | <0.0001 | 3858 | 88%  (68.8 - 97.5) | 60.1%  (55.5 - 64.5) |
| AUC-CSH | 0.52  (0.48 - 0.57) | 0.633 | 0.462 | 51.2%  (35.5 - 6.7) | 58.7%  (54.1 - 3.3) |  | 0.54  (0.43 - 0.65) | 0.464 | 0.453 | 84%  (63.9 - 95.5) | 35.5%  (31.2 - 40.0) |
| GLRLM-RV | 0.66  (0.61- 0.70) | 0.0010 | 0.136* | 16.3%  (6.8 - 30.7) | 97.4%  (95.4 - 98.6) |  | 0.67  (0.63 - 0.71) | 0.0046 | 0.136* | 20%  (6.8 - 40.7) | 97%  (95.1 - 98.4) |

SUVmax, maximum standard uptake value; SUVpeak, peak standard uptake value; MTV, metabolic tumor volume; TLG, total lesion glycolysis; AUC-CSH: area under curve of cumulative SUV-volume histogram; GLRLM-RV, grey-level run length matrix run variance; CI, confidence interval.

*Cut-off points were derived by ROC analysis, except for GLRLM‑RV, which was dichotomised by C‑tree analysis.

.

**Table S5** Multivariable Cox models for progression-free and overall survival

| **Variable** | **HR** | **(95% CI)** | **P-value** |
| --- | --- | --- | --- |
| ***Cox model for PFS***  No. of subjects =495  No. of failures = 43 | | | |
| GLRLM-RV >0.136 | 4.18 | (1.85-9.43) | 0.001 |
| TLG> 3858 | 2.76 | (1.37-5.59) | 0.005 |
| Stage>2 | 4.96 | (1.72-14.31) | 0.003 |
| ***Cox model for OS***  No. of subjects =495  No. of failures = 25 | | | |
| GLRLM-RV >0.136 | 5.25 | (1.98-13.88) | 0.001 |
| TLG> 3858 | 6.99 | (2.02-24.24) | 0.002 |
| Stage>2 | 6.38 | (1.80-22.55) | 0.003 |
| ECOG PS>1 | 3.92 | (1.51-10.14) | 0.005 |

**Table S6.** Univariate analysis of overall survival according to dichotomized baseline clinical and PET parameters (N=501)

| Baseline parameters | No. of  patients | 5-year OS  (95%CI) | HR (95%CI) | P value  (log-rank test) |
| --- | --- | --- | --- | --- |
| Bulk ≤ 100 mm > 100 mm | 145 356 | 99% (95-100%) 94% (91-96%) | 4.48 (1.05-19.04) | **0.0260** |
| Largest lesion diameter ≤ 139.5 mm > 139.5 mm | 368 133 | 97% (95-99%) 90% (83-94%) | 4.35 (1.95-9.68) | **0.0001** |
| Sex Male Female | 199 302 | 94% (90-97%) 96% (93-98%) | 0.61 (0.28-1.33) | 0.2061 |
| Age ≤ 60 yr > 60 yr | 481 20 | 96% (93-97%) 95% (69-99%) | 1.12 (0.15-8.34) | 0.9089 |
| No. of extranodal sites ≤ 1 > 1 | 310 191 | 97% (94-98%) 93% (88-96%) | 2.01 (0.91-4.44) | 0.0774 |
| LDH ≤ ULN > ULN | 146 355 | 96% (91--98%) 95% (92-97%) | 1.04 (0.43-2.48) | 0.9343 |
| Ann Arbor Stage 1-2  3-4 | 489 12 | 96% (94-97%) 75% (41-91%) | 7.99 (2.37-26.94)- | **0.0001** |
| ECOG PS ≤ 1 > 1 | 460 41 | 96% (94-98%) 85% (70-93%) | 3.41 (1.35-8.62) | **0.0059** |
| TLG ≤ 3858 > 3858 | 289 212 | 99% (97-100% 91% (86-94%) | 10.17 (3.04-33.99) | **<0.0001** |
| MTV ≤ 318 ml > 318 ml | 242 259 | 99% (96-100%) 92% (88-95%) | 6.81 (2.04-22.75) | **0.0029** |
| AUC-CSH ≥ 0.453 < 0.453 | 328 173 | 94% (91-96%) 98% (95-99%) | 0.38 (0.13-1.10) | 0.0623 |
| SUVmax ≤ 24.3 > 24.3 | 328 173 | 97% (94-98%) 93% (88-96%) | 2.05 (0.94-4.50 | 0.0664 |
| SUVpeak ≤ 16.1 > 16.1 | 159 342 | 99% (95-100%) 94% (90-96%) | 5.49 (1.29-23.28) | **0.0094** |
| GLRLM-RV ≤ 0.136  >0.136 | 475 20 | 96% (94-98%) 80% (85-92%) | 7.86 (3.14-19.69) | **< 0.0001** |

LDH, Serum lactate dehydrogenase; ULN, upper limit of normal; ECOG, Eastern Cooperative Oncology Group; TLG, total lesion glycolysis; MTV, metabolic tumor volume; AUC-CSH, area under the curve of the cumulative standardized uptake value (SUV)-volume histogram SUVmax, maximum SUV; SUVpeak, peak SUV; GLRLM-RV, grey-level run length matrix run variance; HR, hazard ratio; 95%CI, 95% confidence interval.

**Table S7**. Model performance for progression-free (PFS) and overall survival (OS) prediction. The Ctree-generated PET radiomics model (based on GLRLM run-variance and TLG) from the IELSG37 study cohort is compared with the IELSG26 PET metrics model (based on TLG and AUC-CSH) and with the main DLBCL international prognostic indexes. Performance is evaluated using the concordance probability estimate (Harrell’s C-index) and the Akaike (AIC) and Bayesian (BIC) information criteria.

| Prognostic indices | PFS prediction accuracy | | | OS prediction accuracy | | | |  |
| --- | --- | --- | --- | --- | --- | --- | --- | --- |
|  | **C-index** | **AIC** | **BIC** |  | **C-index** | **AIC** | **BIC** |  |
| IELG37 PET radiomics model | 0.6805 | 490.3 | 494.5 |  | 0.7471 | 268.8 | 273.0 |  |
| Dichotomized GLRLM-RV | 0.5674 | 500.4 | 504.6 |  | 0.5751 | 284.1 | 288.3 |  |
| Dichotomized TLG | 0.6599 | 499.2 | 503.4 |  | 0.7255 | 274.7 | 278.9 |  |
| IELSG26 PET metrics model | 0.6533 | 499.7 | 503.9 |  | 0.6155 | 292.6 | 296.8 |  |
| IPI | 0.5682 | 510.3 | 514.5 |  | 0.5864 | 293.5 | 297.7 |  |
| Revised IPI | 0.5799 | 509.1 | 513.4 |  | 0.6376 | 290.3 | 294.6 |  |
| Age-adjusted IPI | 0.5452 | 496.7 | 500.9 |  | 0.5763 | 279.9 | 284.0 |  |
